# Prevalence and reasons for intentional use of complementary and alternative medicine as an adjunct to future visits to a medical doctor for chronic disease

**DOI:** 10.1186/s12906-018-2179-8

**Published:** 2018-03-27

**Authors:** Agnete E. Kristoffersen, Trine Stub, Frauke Musial, Vinjar Fønnebø, Ola Lillenes, Arne Johan Norheim

**Affiliations:** The National Research Center in Complementary and Alternative Medicine (NAFKAM), Department of Community Medicine, Faculty of Health Sciences, UiT, The Arctic University of Norway, Tromsø, Norway

**Keywords:** Chronic disease, Health care utilization, Complementary and alternative medicine, Norway

## Abstract

**Background:**

Intentional use of complementary and alternative medicine (CAM) has previously only been researched in small, possibly biased, samples. There seems to be a lack of scientific information regarding healthy individual’s attitudes and presumed use of CAM. The aim of this study is to describe prevalence and characteristics of participants who intend to see a CAM provider compared to participants who intend to see a medical doctor (MD) only when suffering from a chronic, non- life-threatening disease and in the need of treatment. Further to describe differences between the groups regarding expected reasons for CAM use and expected skills of CAM providers.

**Method:**

The survey was conducted in January 2016 as part of the “TNS Gallup Health policy Barometer”. In total, 1728 individuals aged 16–92 years participated in the study, constituting an overall response rate of 47%. The survey included questions regarding opinions and attitudes towards health, health services and health politics in Norway.

**Results:**

The majority of the participants (90.2%) would see a MD only if they were suffering from a chronic, non- life-threatening disease and were in the need of treatment. Men over the age of 60 with a university education tended to see a MD only. Only 9.8% of all respondents would in addition visit a CAM provider. Being an intentional user of a MD + CAM provider was associated with being a woman under the age of 60. The respondents believed that CAM providers have professional competence based on formal training in CAM. They also believed that individuals seeing a CAM provider have poor health and are driven by the hope of being cured. Further, that they have heard that others have good experience with such treatment.

**Conclusion:**

Intentional use of CAM is associated with positive attitudes, trustworthiness, and presumed positive experiences in the CAM-patient-setting. Intentional CAM users also have the impression that CAM providers have professional competence based on formal training in alternative therapies.

**Electronic supplementary material:**

The online version of this article (10.1186/s12906-018-2179-8) contains supplementary material, which is available to authorized users.

## Background

Complementary and alternative medicine (CAM) consists of a group of diverse medical and health care systems, practices and products that are not considered to be a part of conventional healthcare. These interventions are mostly used in conjunction with (complementary to) conventional health care [1].

The use of CAM in general and the use of a CAM provider more specifically is widely used both worldwide, in Europe and in Norway [2–4], ranging from 2% to 49% 12-month prevalence [5, 6].

The typical user of a CAM provider is a well-educated young to middle-aged women [3–5, 7] who visits a CAM provider for various reasons: A desire to achieve a more holistic treatment, to take active part in the treatment [8, 9], because she trusts the CAM providers [8, 10] or because she has distrust in conventional care [11]. She might have a desire to use natural products with minimal adverse effects to remain in good health and improve quality of life. Family, friends or her medical doctor (MD) might have recommended the CAM treatment [12].

The definition of CAM varies widely: from the utilization of a CAM provider to the inclusion of over-the counter products, CAM techniques, special diets, exercise and prayer [13]. While over-the-counter products, CAM techniques, special diets, exercise and prayer might be administrated by the patient him- or herself, visits to a CAM provider always include a trained therapist.

In Norway CAM providers are defined as providers other than authorized health professionals who give health-related treatment that mainly is offered outside public health care [11]. There are no educational requirements to legally practice as a CAM provider in Norway [14, 15] in contrast to authorized health professional who needs a public approval/license to practice. The most commonly used CAM providers offer massage, acupuncture, reflexology and spiritual healing [13, 16]. Twelve months prevalence of using a CAM provider in Norway is reported to be 13–24% [3, 4, 17].

All Norwegian citizens are provided with a regular MD that offers medical services in primary care. Visits to the MD are co-paid with a small fee [13] in contrast to visits to CAM providers that are fully covered by the patients themselves. Approximately 82% of all Norwegians see an MD during 1 year [7], 13% of these also see a CAM provider [7].

The contextual and sociocultural factors influencing CAM use are still not well understood. Patient surveys are often selective and the representativeness of the target group is often questioned. One of the obstacles is that most studies reporting prevalence and epidemiological data are undertaken in patients within the conventional healthcare system. Already being a CAM user might bias survey results even more.

Intentional CAM use has previously only been researched in small, possibly biased samples [8]. There seems to be a lack of scientific information regarding healthy individual’s attitudes and presumed use of CAM. Surveying healthy individuals’ intentional use, may contribute to a deeper understanding of the population’s attitudes towards CAM.

In this study, the intentional use of a CAM provider as an adjunct to an MD, are compared to intentional use of MD only.

The aims of the study presented here are to describe the:

1. Prevalence and characteristics of participants who intend to see a CAM provider in addition to an MD compared to participants who intend to see an MD only when suffering from a chronic, non- life-threatening disease and in the need of treatment.
2. Describe differences between the two groups regarding:
  - The participants expected reasons for why individuals see a CAM provider
  - What skills and qualifications the study participants expect the CAM providers to have.

## Methods

TNS Gallup is a Norwegian commercial group conducting interview-based marketing analyses for different stakeholders. TNS Gallup is part of the market research company Kantar TNS.

The current web based survey was conducted in January 2016 as part of the *TNS Gallup Health policy Barometer*, a population-based survey focusing on health-related issues derived from different stakeholders engaged in commercial interests, idealistic work and research institutions. With regard to attitudes to, and intentional use of CAM, the questions were developed at the Norwegian National research center in complementary and alternative medicine (NAFKAM) for this study. See Additional file 1 for a detailed description of the questions.

TNS Gallup has an access panel for surveys consisting of about 45,000 individuals who have given consent to participate and who regularly answering surveys. Among these, a sample of 3702 potential participants were selected for this survey.

This TNS Gallup panel is put together in terms of representativeness reflecting the Norwegian population. Members of the panel are mainly recruited through representative surveys conducted by telephone, using probability sampling. This sampling method implies that every member of a population has a chance (greater than zero) of being selected in the sample. This probability can be accurately determined by weighting sampled units according to their probability of selection.

The sample used in the TNS Gallup Health policy Barometer was stratified by gender, age and geography. After data collection, data were weighted to correct for random sampling error and non-response. The sample was weighted by gender, age, geography and highest level of education.

**Fig. 1:**
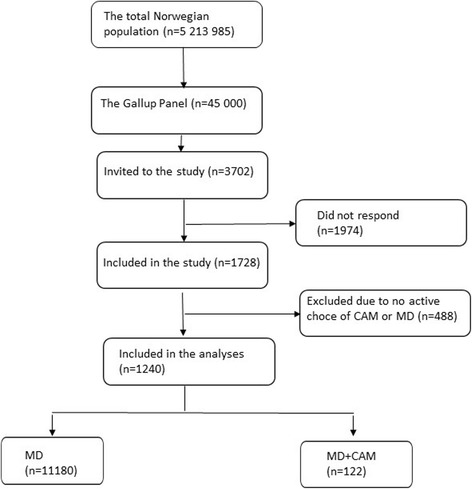
Flow chart showing the selection of the studied population

In total, 1728 individuals 16–92 years of age participated in the study, constituting an overall response rate of 47% representing 39–50% of the stratified groups. Four hundred and eighty-eight were excluded in the analysis presented here, because they did not provide an explicit choice of an MD or MD + CAM provider, resulting in 1240 included participants in the analysis (Fig. 1).

The survey included questions regarding opinions and attitudes towards health, health services and health politics in Norway. Participants indicated their consent to participate in this particular study by answering and returning the questionnaire. The questions concerning CAM were focused around intended use of CAM, the participants’ expectations with regard to skills of CAM providers, expected reasons for visiting a CAM provider, and opinions about reimbursement of CAM providers’ fees.

An intentional MD user was defined by agreeing to the statement; *“I usually see only a medical doctor”* to the question *“If you have a chronic, but not life-threatening disease and need treatment, which of the following alternatives describes you best?”*

An intentional MD  + CAM user in this study was defined by agreeing to the statement; *“I seek usually both a medical doctor and a CAM provider.”* to the same question. These respondents answering MD + CAM are labelled intentional MD +  CAM users in the study.

The respondents answering either *“What I do depends on the type of illness, situation or where I am”*, *“I usually go to a CAM provider rather than to a medical doctor”,* “*I seek usually neither a medical doctor nor a CAM provider”* or “*Don’t know”* were excluded from the analyses (Fig. 1).

Between-group differences were analyzed using chi-square tests for binary data analyzing one variable at the time in SPSS for Windows (version 22.0, SPSS, Inc., Chicago, IL). Significance level was defined as *p* < 0.05 without *p*-value adjustment for multiple comparisons.

## Results

Of 1240 presumed healthy respondents who had explicitly indicated a choice regarding intentional use of MD and/or a CAM provider, 1118 (90.2%) indicated that they usually only would see an MD if they had a chronic, but not life-threatening disease, while 122 respondents (9.8%) indicate that they would see both an MD and a CAM provider (Fig. 1).

### Basic characteristics of participants

When comparing intentional MD + CAM users with the MD users, we found that women (*p* < 0.001), and respondents under the age of 60 (*p* = 0.034) to a higher degree intended to add CAM to their MD treatment. Men, individuals over the age of 60, and individuals with university education intended to a higher degree to only see an MD.

Neither place of residence (rural/city), household income nor education was associated with being an intentional MD + CAM user or intentional MD user (Table 1).

**Table 1:** Basic characteristics of participants

	MD		MD + CAM		*p*-value
	%	n (1118)^a^	%	n (122)^a^	
Gender					< 0.001
Men	53.7	(600)	36.1	(44)	
Women	46.3	(518)	63.9	(78)	
Age					0.034
Less than 30 years of age	10.1	(113)	13.9	(17)	
30–44 years	20.5	(229)	23.0	(28)	
45–59 years	25.6	(286)	32.8	(40)	
60 years or more	43.8	(489)	30.3	(37)	
Place of residence					0.908
Rural	89.0	(995)	89.3	(109)	
City	11.0	(123)	10.7	(13)	
Education					0.099
Low to middle	43.0	(481)	50.8	(62)	
High (university)	57.0	(637)	49.2	(60)	
Household income					0.642
Low	35.1	(356)	31.5	(34)	
Middle	41.8	(423)	46.3	(50)	
High	23.1	(234)	22.2	(24)	

^a^ Due to missing responses the numbers do not always add up to the total number

The health-related measurements “*healthiness of diet*”, “*self-reported health*”, “*physical activity*” and “*body mass index (BMI*)” based on reported height/weight was equally distributed in the two groups.

### Expected skills of the CAM providers

When asked about expected skills of CAM providers, the intentional MD + CAM users more often than intentional MD users expected them to have “*professional competence based on formal training in CAM therapies”,* (72.1% and 33.6%, respectively, *p* < 0.001, Table 2-Q3). On the other hand, intentional MD users more often expected CAM providers to have “*no special skills”* (31.2% and 4.9% respectively, p < 0.001, Table 2-Q5). A minor proportion of both groups held the view that CAM providers had “*no professional skills but special healing abilities”* (10.9% and 6.6%, respectively, *p* = 0.136, Table 2-Q4).

**Table 2:** Expected skills of CAM providers^a^

	MD		MD + CAM		*p*-value
	%	n (1118)	%	n (122)	
What skills do you think most CAM providers have?					
1. *Authorized as health personnel*	4.7	(52)	7.4	(9)	0.186
2. *Knowledge of the body at the level of nurses*	7.4	(83)	9.8	(12)	0.342
3. *Professional competence based on formal training in alternative therapies*	33.6	(376)	72.1	(88)	< 0.001
4. *No professional skills but special healing abilities*	10.9	(122)	6.6	(8)	0.136
5. *No special skills*	31.2	(349)	4.9	(6)	< 0.001
6. *Don’t know*	18.1	(202)	9.0	(11)	0.012

^a^ Multiple answer possible regarding statement 1–3

Neither intentional MD users, nor intentional MD + CAM users expected CAM providers to be “*authorized health care personnel”* (4.7% and 7.4%, *p* = 0.186, Table 2-Q1), or to have “*knowledge at the level of nurses”* (7.4% and 9.8%, *p* = 0.342, Table 2-Q2). Eighteen per cent of intentional MD users did not know what skills CAM providers would have, compared to 9.0% of the intentional MD+ CAM users (*p* = 0.012, Table 2-Q6).

### Reasons for CAM use

The most commonly reported expected reason for an individual’s CAM use was because “*They were not healthy and driven by the hope of being cured”* (*n* = 661, Table 3-Q8), followed by “*Others having good experience with such treatment”* (*n* = 514, Table 3-Q2) and “*Because they were open to try whatever”* (*n* = 471, Table 3-Q7). Few respondents believed that “*Research has shown that treatment works”* (*n* = 196, Table 3-Q1).

**Table 3:** Expected reasons for CAM use ^a^

	MD		MD + CAM		*p*-value
	%	n (1118)	%	n (122)	
What do you think are the main reasons that individuals are seeing a CAM provider?					
*1. They believe that research has shown that treatment works*	6.6	(74)	18.0	(22)	< 0.001
*2. They have heard that others have good experience with such treatment*	39.7	(444)	57.4	(70)	< 0.001
*3. They believe that CAM providers spend more time with patient*	7.9	(88)	17.2	(21)	0.001
*4. They believe that the CAM provider have a more holistic view than conventional health care personnel*	20.0	(224)	42.6	(52)	< 0.001
*5. They believe that CAM therapies are more natural and harmless than conventional therapies*	19.9	(223)	24.6	(30)	0.227
*6. They have bad experiences with public health care*	29.5	(330)	30.3	(37)	0.852
*7. They are generally open to try what ever*	37.6	(420)	41.8	(51)	0.360
*8 They are not healthy and driven by the hope of being cured*	54.4	(608)	43.4	(53)	0.021
*9.Don’t know*	9.5	(106)	2.5	(3)	0.009

^a^ Multiple answers possible

The main differences between intentional MD users and intentional MD + CAM users was found regarding *“Research has shown that treatment works*” (6.6% versus 18%, *p* < 0.001, Table 3-Q1)), “*CAM providers spend more time with their patients”* (7.9% versus 17.2%, *p* = 0.001,Table 3-Q3), “*CAM providers have a more holistic view”* (20% versus 42.6%, *p* < 0.001, Table 3-Q4) and “*That others have good experiences with such treatment”* (39.7% versus 57.4%, *p*  < 0.001, Table 3-Q2).

Intentional MD users were more likely than intentional MD + CAM users to expect CAM users to be “*Not healthy and driven by the hope of being cured”* (54.4% versus 43.4, *p* = 0.021, Table 3-Q8).

## Discussion

### Key findings

The majority of the participants (90.2%) intend to see only an MD assuming they should suffer from a chronic, not life-threatening disease and were in the need of treatment. Only a small proportion (9.8%) would also see a CAM provider. Being an intentional CAM user was associated with being a woman and younger than 60 years old. These respondents believe that CAM providers have professional competence based on formal training in CAM and that individuals seeing a CAM provider are not healthy and driven by the hope of being cured.

### Bias consideration

In this study, the intentional use of a CAM provider, is surveyed in a questionnaire which includes other non-related questions developed by many other interests and contributors. The responses with regard to potential use of CAM might therefore depend on opinions, attitudes, feelings and beliefs towards other aspects and interest areas. The content and order of questions might also influence the responses, as the internal validity of the questionnaire as a whole has not been scrutinized specifically. However, the TNS Gallup methodology has been in common use for many years. The large cohort and the relatively high response rate also ensure the content validity of the questionnaire.

The focus in this paper is healthy individuals’ intentional use of CAM. The phrasing of the question could also, in part, be regarded as being based on previous healthcare experience. The term “*I usually go to*….” might imply that the responses rely on earlier experiences with health care. The situation description is, however, prospective, and responses to the question must therefore be seen as indicative of intentional use in the future.

The variable *intention to use,* is associated with some limitations. What people say they intend to do if they should be ill in the future is often different from what they actually do when they become ill. *Optimistic bias* refers to the phenomenon that a person claims to be less at risk than others, even though the general risk constellation is similar [18]. Studies of several specific health and safety hazards suggest that individuals tend to believe that their own risk is below average, demonstrating an optimistic bias.

This phenomenon has been shown for a variety of health risks and is not dependent on age, gender, education or occupation [18]. In health psychology and behavioural medicine “optimistic bias” or “unrealistic optimism” in risk perception is a major hindrance for the prevention of disease. Since risk perception is related to behaviour, “optimistic bias” may impede a person to adhere to screening procedures or to induce health protective life-style changes [19, 20]. The influence of the optimistic bias could have led to a lower presumed use of CAM in this study compared to actual use when facing a health problem.

### Prevalence and associations for use

As might be expected, considering the phenomenon of “optimistic bias”, 9.8% intentional use of a CAM provider if suffering from a chronic, not life-threatening disease, is slightly lower than what was found in a Norwegian study finding that 13.3% of the participating seeing an MD last year also saw a CAM provider [7, 16].

The higher presumed use of a CAM provider among women and individuals at a younger age, is in line with previous findings in individuals already using CAM providers [3, 4, 21]. The higher use in these groups might be due to the fact that CAM is a rather new approach to health care in the Western world and that women and younger individuals tend to be more open to adapt to new systems than men and older individuals who tend to be more traditional in their choice [22]. The nature of CAM might also be seen as more in line with the feminine role than the masculine, more mechanical, view of the body [3].

The similarity of the two studied groups regarding “education”, “household income”, “place of residence”, “healthiness of diet”, “self-reported health”, “physical activity” and “BMI” shows that the individuals intending to add a CAM provider to their future health care choice, is similar to those not intending to make that choice in most respects except gender and age. This is in line with previous research [2] showing that CAM use is no longer a phenomenon restricted to a unique segment of the population that is highly educated and/or has a high family income [20, 21].

The intentional use of CAM is, however, lower than predicted use of CAM found in a comparable Israeli study where 54% of the respondents replied that they intended to use CAM within the next year. Similarities were found with regard to the fact that predicted future use was similar to reported use within the past year [10]. Intention of future use is considered one of several indications of commitment to a specific treatment modality and is often based on earlier experiences with this kind of treatment [23].

### Expected skills and qualifications of the CAM providers

As described earlier, there are no educational requirements to legally practice as a CAM provider in Norway [14, 15]. The professional associations of CAM providers, however, do normally require CAM education at a certain level, and sometimes also conventional medical education, for membership in the association. A membership in an association is needed to register in the voluntary official register for CAM providers giving financial advantages to the CAM providers and their patients [24].

The highest proportion of respondents in both groups presume that CAM providers have “*Professional competence based on formal training in CAM therapies”*. This is in line with the requirement of most of the CAM associations and therefore also the requirement of the official register for CAM providers in Norway.

The majority of intentional CAM users see CAM providers as professionally qualified. The reason a lower proportion of intentional MD users seeing CAM providers as professionally qualified, could be due to low effort in searching this knowledge about CAM providers, as they do not intend to use this service themselves. Assuming a low level of competence among CAM providers might also justify their choice of not intending to add CAM treatment to their future use of an MD. The high proportion of the total study population that did not expect CAM providers to be *authorized health care personnel* or have *medical knowledge at the level of nurses*, shows that respondents are fairly knowledgeable with regard to CAM providers’ educational level.

The spiritual healer association in Norway requires only documented healing skills for membership and not formal education in healing. These CAM providers might be in mind for the 130 respondents (122 + 8, Table 2, Q5) holding the view that CAM providers have no professional skills but special healing abilities.

The high proportion of the total study population that did not expected CAM providers to be *authorized health care personnel* or have *medical knowledge at the level of nurses*, shows that the actual knowledge of the CAM provider’s skills is well known among the participants. This point of view undergird the thoughts that the population expect CAM providers to work outside, and different from what is done within the conventional health care system. On the other hand - the fact that some respondents also presume that CAM providers have medical training at the level of nurses, might be interpreted as the CAM field is on its way to be trustworthy in terms of medical qualifications. The higher proportion of CAM users holding this view could be explained by the fact that many CAM providers are also authorized health care personnel practicing CAM both outside and within conventional health care settings [16, 25].

### Expected reasons for seeing a CAM provider

The most commonly reported assumed reason for CAM use was “*They* [the patients] *were not healthy and driven by the hope of being cured”* (*n* = 661, 53.3%). This is somewhat higher than what is found in studies mapping cancer patient’s reasons for CAM use [26]. This expected reason was found more frequently among the intentional MD users, and might be an expression for a view where CAM use is seen as a desperate, unrealistic search for a cure more than real healthcare action. On the other hand, intentional CAM users might be more realistic about what to expect from CAM treatment.

The reason “*They are generally open to try whatever”* is reported by nearly 40% of participants in both groups. These findings are in accordance with a recent study on German cancer patients, finding that approximately 45% reported the reason for CAM use to be *“To not miss any chance*” [27]*.*

Most of the intentional CAM users (57.4%) reported “*Others having good experience with such treatment”* as the expected reason for CAM use. The result underpins the idea that *word of mouth* is an important source of information for the decision of adding CAM to the treatment. This reason is found also in other studies, however less frequently [27, 28]. The intentional MD users might have lower confidence in other patients’ experience as they report “*Others having good experience*” less frequently than the intentional CAM users.

## Conclusions

The majority of the participants intend to see an MD only, if suffering from a chronic, not life-threatening disease. Only a small proportion would in addition see a CAM provider. Intentional use of CAM is associated with positive attitudes, trustworthiness, and presumed positive experiences in the CAM-patient-setting. Intentional CAM users also have the impression that CAM providers have professional competence based on formal training in alternative therapies. This study shows that a similar pattern of prevalence of use and attitudes towards CAM found in the general population, is also found with regard to intentional CAM use in healthy individuals. Our results might, with great caution, be introduced into the debate of who are stakeholders for priorities in the general healthcare debate; the patients or the healthy people? According to our results, healthiness does not disqualify a healthy person from having the viewpoint of the patient in mind.

## Additional file

Additional file 1: The questions used in this study. (DOCX 17 kb)

## Abbreviations

BMI: Body mass index
CAM: Complementary and alternative medicine
MD: Medical doctor
TNS: Taylor Nelson Sofres
