# Supplementary material for: Prevalence and reasons for intentional use of complementary and alternative medicine as an adjunct to future visits to a medical doctor for chronic disease
Source: BMC Complement Altern Med. 2018 Mar 27;18:109. doi: 10.1186/s12906-018-2179-8 (PMC5870092; doi:10.1186/s12906-018-2179-8)
Supplement: Supplementary file 1 — The questions used in this study. (DOCX 17 kb) [file 12906_2018_2179_MOESM1_ESM.docx]

1. If you have a chronic, but not life-threatening disease or complaint that needs treatment, which of these statements best describes you? Choose only one alternative.

1 I usually consult a CAM provider rather than a GP.

2 I usually consult both a GP and a CAM provider.

3 I usually consult only a GP.

4 I usually don´t consult GPs or CAM providers, but I buy dietary supplements, herbs or similar natural products made for self treatment.

5 I usually don´t consult GPs or CAM providers, but I buy over-the-counter products made for self treatment.

1. What I do, depends on the type of disease, the situation, or where I am.

999 Don´t know.

1. When you think of the majority of the CAM providers who work in Norway, what kind of education and experience do you think they have? You may choose multiple alternatives.

1 Qualified health personnel.

2 Knowledge of the body, similar to that of a nurse.

3 Expertise based on formal education in complementary and alternative treatment.

4 No expertise, but special healing abilities (warm hands and clairvoyance).

1. No special expertise.

999 Don´t know.

1. If you were to buy dietary supplements, vitamins or herbal medicines, what would be important to you? You may choose multiple alternatives.

1 The product must be approved by the health authorities.

2 The product must be recommended by the GPs or drugstores.

3 The product must be recommended by CAM providers.

4 The product must be recommended by health food stores.

5 The product must be recommended through advertising.

6 The product must be recommended by friends or family.

7 The product must be provided with a list of ingredients.

8 I buy these products independent of other people´s opinion.

9 I don´t buy such products.

10 Other issues.

999 Don´t know.

1. What are the most important reasons for people to consult CAM providers, do you think? You may choose multiple alternatives.

1 They believe that research has shown that this treatment works.

2 They have heard that others have benefited from such treatment.

3 They believe that CAM providers spend more time with their patients.

4 They believe that CAM providers are better at seeing the ”whole” patient.

5 They believe that CAM modalities are more natural and harmless than conventional medicine.

1. They have bad experiences with the health services.
2. They are generally more open-minded about trying ”everything”.
3. They aren´t getting well, but are driven by the hope of being cured.

9998 None of the above.

9999 Don´t know.

1. Do you think that the national insurance should cover people´s expenses for CAM treatment? This means that you would only have to pay a small amount, or does this kind of expenses belong outside the public economy? Choose only one alternative.

1 You should only pay a small amount of your expenses for CAM treatment, like you do for ordinary health services.

2 CAM treatment expenses should not be covered by the national insurance.

9999 Don´t know.
